# Infection and inflammation

**DOI:** 10.1186/1471-2393-12-S1-A8

**Published:** 2012-08-28

**Authors:** James A McGregor, Janice I French, Marti Perhach

**Affiliations:** 1 LA Best Babies Network, Los Angeles, CA, USA; 2 Group B Strep International, Pomona, CA, USA

## 

Potentially preventable maternal infections are increasingly recognized as being a cause of fetal death or stillbirth. McClure and Goldenberg maintain that infection causes 20% to 40% of fetal deaths in developed and developing countries respectively [1]. Infection causes embryonic, fetal, and perinatal death by multiple mechanisms including:

1) direct, blood borne placental infection (via maternal blood stream),

2) ascending vaginal infection through the cervix,

3) iatrogenic infection (such as amniocentesis and “membrane stripping”), and

4) persistent uterine infection already present during implantation [2].

More recently recognized mechanisms include:

1) combined viral and microbial infections, in which placental viral infection sensitizes to the effects of bacterial lipopolysaccharide [3],

2) “graft vs. host” immunologic phenomena in which ongoing or preceding infections may potentiate maternal and/or fetal inflammatory rejection mechanisms, and

3) “sterile inflammation” considered to be due to innate immune or “autophagic” mechanisms which are required to remove dead cells caused by “senescence” or apoptosis [4].

The types of microbes causing stillbirth vary by location, population, and occupational factors. These include all taxa of microbes: bacteria, mycoplasmas, viruses (entero and herpes viruses), fungi (yeasts), rickettsia (Q-fever and Rocky Mountain spotted fever), parasites (toxoplasmosis), and possibly nematodes and transposons or prions.

Infection-caused stillbirth or septic miscarriage occurs when invasive microorganisms either overwhelm embryonic or fetal innate and adaptive immune host defense mechanisms or cause sufficient cell and tissue death to result in the death of the entire placenta and embryo or fetus. Less extensive intrauterine infections or inflammation can also damage the developing embryo or fetus causing lifetime disability. We now understand that host immune cells and tissue pathophysiologic “death mechanisms” can stimulate necrosis (death of internal cell organelles) or apoptosis (due to “death programming”) cell signalling [5]. Both processes may cause considerable organ damage in vulnerable organs, especially the central nervous system and special sense organs (retina, hearing organs) without causing “cardiac death” clinically recognizable by loss of heartbeat. As an example, Group B streptococcus (GBS) is widely recognized to cause lifelong brain injury from meningoencephalitis or systemic “FIRS” (fetal inflammatory reaction syndrome.) Infection-caused stillbirth may occur as isolated cases (syphilis) or in epidemics (Q-fever or foodborne Listeria infection.)

Successful programs prevent prevalent causes of infection-caused stillbirth depend upon:

1) recognition of the problem,

2) means to identify the agent, and

3) effective means to treat intrauterine infections.

Prevention of GBS infection is an example of programmatic primary prevention of septic stillbirth through identification and treatment of GBS urinary tract infection or asymptomatic bacteriuria with penicillin followed by test of cure (TOC) [6].

Other successful examples of secondary prevention are anti-syphilis and anti-HIV programs which unite maternal screening and indicated treatments. Primary prevention (before maternal infection) of infection-causing reproductive loss depends on the knowledge of the epidemiology and pathophysiology of the particular infectious agents. Examples include preventing rubella and varicella by vaccinations, listeriosis via milk pasteurization, and malaria by using mosquito netting. Prevention of more recently understood causes, such as ascending *in utero* infection due to GBS, BV, or associated vaginal microflora, will require new care practice strategies including expanded screening and maternal vaccination. Such prenatal programmatic change will require a concerted and organized effort.

In Germany, weekly vaginal pH screening using a *sensitive* indicator (increased vaginal pH over 4.7) has been used to indicate the need for *specific* microbial testing and treatment. Other approaches to preventing ascending vaginal infection include specific screening for prevalent abnormal microflora (vaginitis, GBS, or sexually transmitted infections) in populations at risk.
